# Speech Processing as a Far-Transfer Gauge of Serious Games for Cognitive Training in Aging: Randomized Controlled Trial of Web-Based Effectivate Training

**DOI:** 10.2196/32297

**Published:** 2022-07-28

**Authors:** Gal Nitsan, Shai Baharav, Dalith Tal-Shir, Vered Shakuf, Boaz M Ben-David

**Affiliations:** 1 Department of Communication Sciences and Disorders University of Haifa Haifa Israel; 2 Baruch Ivcher School of Psychology Reichman University (IDC) Herzliya Israel; 3 Department of Communications Disorders Achva Academic College Arugot Israel; 4 Toronto Rehabilitation Institute University Health Networks Toronto, ON Canada; 5 Department of Speech-Language Pathology University of Toronto Toronto, ON Canada

**Keywords:** aging, cognitive aging, cognitive games, serious games, speech processing, spoken language processing, eye tracking, visual world paradigm

## Abstract

**Background:**

The number of serious games for cognitive training in aging (SGCTAs) is proliferating in the market and attempting to combat one of the most feared aspects of aging—cognitive decline. However, the efficacy of many SGCTAs is still questionable. Even the measures used to validate SGCTAs are up for debate, with most studies using cognitive measures that gauge improvement in trained tasks, also known as *near transfer*. This study takes a different approach, testing the efficacy of the SGCTA—*Effectivate*—in generating tangible *far-transfer* improvements in a nontrained task—the Eye tracking of Word Identification in Noise Under Memory Increased Load (E-WINDMIL)—which tests speech processing in adverse conditions.

**Objective:**

This study aimed to validate the use of a real-time measure of speech processing as a gauge of the far-transfer efficacy of an SGCTA designed to train executive functions.

**Methods:**

In a randomized controlled trial that included 40 participants, we tested 20 (50%) older adults before and after self-administering the SGCTA *Effectivate* training and compared their performance with that of the control group of 20 (50%) older adults. The E-WINDMIL eye-tracking task was administered to all participants by blinded experimenters in 2 sessions separated by 2 to 8 weeks.

**Results:**

Specifically, we tested the change between sessions in the efficiency of segregating the spoken target word from its sound-sharing alternative, as the word unfolds in time. We found that training with the SGCTA *Effectivate* improved both early and late speech processing in adverse conditions, with higher discrimination scores in the training group than in the control group (early processing: *F*_1,38_=7.371; *P*=.01; *η*_p_^2^=0.162 and late processing: *F*_1,38_=9.003; *P*=.005; *η*_p_^2^=0.192).

**Conclusions:**

This study found the E-WINDMIL measure of speech processing to be a valid gauge for the far-transfer effects of executive function training. As the SGCTA *Effectivate* does not train any auditory task or language processing, our results provide preliminary support for the ability of *Effectivate* to create a generalized cognitive improvement. Given the crucial role of speech processing in healthy and successful aging, we encourage researchers and developers to use speech processing measures, the E-WINDMIL in particular, to gauge the efficacy of SGCTAs. We advocate for increased industry-wide adoption of far-transfer metrics to gauge SGCTAs.

## Introduction

### Background

The age distribution of the world’s population is projected to dramatically shift over the next few decades as improved health care continues to extend life expectancy [1]. By 2050, more than one-fourth (27%) of the European population is expected to be aged >65 years. Although medicine can prolong relative physical health [2], offsetting age-related changes in cognitive health is growing in importance [3]. Although recent literature suggests that cognitive measures may inflate age-related decreases in performance [4-6] and disregard an increase in crystallized intelligence (eg, general knowledge and vocabulary [7]), a decrease in cognitive performance is one of the most feared aspects of aging [8]. Consequently, there is growing pressure to prolong active and healthy aging—*successful aging* [9]—by targeting age-related changes in cognitive abilities such as memory and executive functions (EFs; eg, inhibition and working memory [WM]) [10,11].

Numerous serious games for cognitive training in aging (SGCTAs) are being developed to mediate age-related cognitive changes. However, there is much debate in the literature regarding their efficacy. In fact, in 2014, a total of 2 teams of researchers published contradictory open letters. The first letter by a group of 70 scientists refuted the efficacy of such training [12]. The second letter by another group of 133 scientists claimed the opposite, supporting the benefit of cognitive training [13]. These letters were followed by an extensive review [14] suggesting that SGCTAs, in general, can improve performance on the trained game and associated activities—*near transfer*. However, the review cautions that there is insufficient evidence to suggest that these changes can *generalize* to activities that are not directly associated with the game—*far transfer*. As cognitive training games and interventions are a “means to enhance performance on other tasks” [14], it seems critical to measure their effects using far-transfer measures that gauge daily activities. Such far-transfer measures would gauge cognitive abilities through performance on a different task that is mediated by the trained cognitive functions.

Effective communication and speech perception play an extensive role in many daily activities and have evident effects on general health and well-being [15]. Difficulty in understanding speech in adverse conditions (eg, noisy background or while conducting another task) forms one of the most prevalent complaints among older adults [16]. These difficulties decrease the participation of older adults in social and professional interactions, thus limiting their independence and increasing feelings of loneliness. Growing evidence suggests that a decrease in speech processing, in turn, has a negative effect on mental health, general well-being, and even longevity [17-21]. The social restrictions imposed by the COVID-19 pandemic further limit interactions and other opportunities for cognitive exercise (eg, work and volunteering). Indeed, current restrictions have been found to increase loneliness and depression in older age [22], even after vaccinations were made available [23] and severe social restrictions were lifted [24]. These, together with limited access to health care services as a result of the pandemic [25,26], illustrate the necessity to create effective SGCTAs that can directly affect spoken communication in adverse conditions, even while social distancing. We suggest that testing speech processing as a far-transfer task could demonstrate the impact of training with SGCTAs, on the daily lives of older adults.

In this exploratory study, we used an eye-tracking paradigm to assess whether training EFs with the SGCTA *Effectivate* generalizes to improved speech processing in adverse listening conditions for older adults. This will serve to validate a real-time measure of speech perception—Eye tracking of Word Identification in Noise Under Memory Increased Load (E-WINDMIL)—as a gauge of far-transfer efficacy of SGCTAs designed to train EFs and provide a case study for developers and academics on the use of far-transfer metrics.

### EFs and Speech Processing

Many SGCTA developers are targeting EFs because of their prominent role in healthy cognitive aging (refer to the seminal work by Salthouse [27,28]). EFs, which include WM and inhibition, enable active maintenance and manipulation of bottom-up information with top-down information in memory, especially during the performance of a concurrent task [29-31]. The literature suggests that individuals with better EFs are able to hold more incoming information and incorporate and manipulate it more easily, even under adverse conditions such as distractions (ie, external noise) and memory preload (ie, remembering the context of a conversation [32,33]). Therefore, it is not surprising that EFs play a significant role in speech processing [34].

Consider a scenario in which an older adult is driving his grandson in a car and radio music is playing. The grandson says, “grandpa, have you seen the DOLL?” The older listener must perform the following tasks:

1. Segregate the spoken message from the background radio noise (task-irrelevant) stream as it unfolds in time
2. Inhibit the activation of competing (similar-sounding) words in the mental lexicon (eg, *DO*/ sounds in words such as *DOG*) while increasing the activation of the word DOLL, as the sound *L* unfolds in time
3. Allocate enough resources for the activities mentioned previously from a limited cognitive resource pool that is already depleted by the concurrent task of driving

As mentioned previously, EFs, especially WM, are essential to perform this complex task and have been shown to be affected by aging in the following ways: (1) stream segregation slows with aging, (2) decrease in the efficiency of inhibition impairs the ability to reject incorrect lexical candidates, (3) decrease in cognitive resources can impair speech perception, and (4) age-related hearing loss distorts the perception of bottom-up signals.

First, stream segregation slows with aging [35]. Reduced WM capacity has been linked to limitations in inhibition [36]. This affects the ability to separate relevant speech from irrelevant background noise. For example, in a study by Janse [37], when speech was presented in background noise, poor inhibitory abilities led to greater interference by the competing noise, which impaired speech perception in older adults.

Second, an age-related decrease in the efficiency of inhibition [15,38] impairs the ability to reject incorrect lexical candidates as the context unfolds in time [39,40].

Third, age-related decreases in cognitive resources, specifically in EFs and WM [38], can impair speech perception, as suggested by the *Framework for Understanding Effortful Listening* [41]. The *Framework for Understanding Effortful Listening* is an adaptation of the capacity model of attention by Nobel laureate Daniel Kahneman, which conceptualizes the relationship between mental resource capacity and cognitive demands. According to this model, mental resources have limited capacity. The presence of background noise or another resource-consuming task (eg, driving) can impede and slow down speech processing for people with lower resource capacities.

Finally, age-related hearing loss distorts the perception of bottom-up signals, providing impoverished input to the central nervous system. To mitigate these effects, older adults rely heavily on the linguistic context in word recognition, often to an even greater degree than younger adults [34,42,43]. Efficient context processing depends on WM capacity and information processing speed [44]. As mentioned previously, these capabilities decline with age [28,45] and can affect older adults’ ability to use context during word recognition. Depleted WM capacity can also affect the ability to temporarily remember words from a given linguistic context for later use [46].

In summary, cognitive performance is intertwined with speech perception, especially in older age. Age-related difficulties in speech perception are not only affected by reduced cognitive abilities but can also accelerate the rate of cognitive decline. A total of 2 Lancet reports [15,47] on dementia prevention highlighted improving auditory and speech accessibility as the number one modifiable risk factor in middle to late life. In fact, the relative weight of speech accessibility in preventing dementia is estimated to be higher than in tackling smoking, diabetes, hypertension, and obesity altogether. As Lin [48] suggests in his *Aging and Cognitive Health Evaluation in Elders* *model* [49], degraded speech processing affects cognitive resilience in aging by decreasing physical activities, social interactions, communication, and related brain functions. Hence, it is plausible to assume that training EFs should enable participants to juggle informational weight more gracefully and process speech faster, in turn, improving their quality of life and well-being.

### Speech as a Far-Transfer Measure of Cognitive Training Using Eye Tracking

To test the effect of cognitive training on speech processing, this study used eye tracking. We used a noninvasive infrared light source and high-precision camera that collects reflections from the eye and records the exact location of the eye gaze on the display at a rate of 500 samples per second. As the word unfolds in time, eye gaze data are time locked with what is being heard by the listener. By recording the participant’s eye movements in relation to the visual display and auditory stimuli, eye tracking provides a highly sensitive and continuous measure of spoken word processing. Unlike overt non–real-time responses (participant verbally or physically responding *after* the word has been heard), the covert rapidity of an eye movement allows one to determine the point in time at which the listener is able to isolate the target word from its competitors through the difference in fixations on the target and competitor over time. Although non–real-time responses, such as pointing at the screen, may be affected by age-related motor slowing, covert eye movement speed and accuracy are relatively unaffected [50].

**Figure 1:**
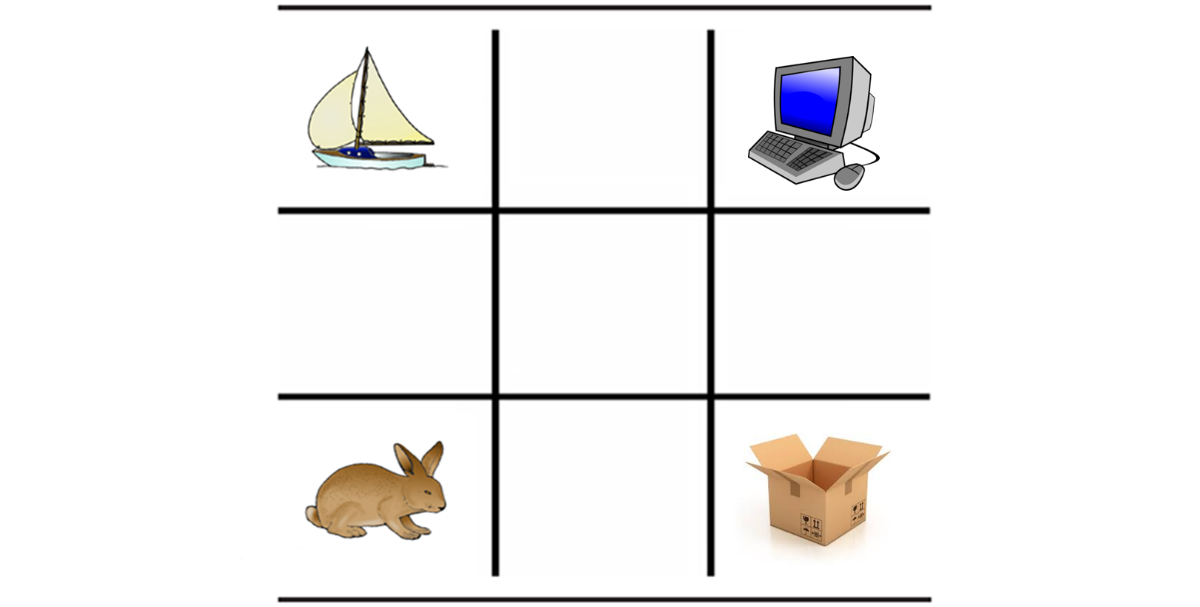
An example of the experimental display. The target word in this example, /ar.nav/ (rabbit), is represented in the bottom-left corner. The phonological competitor, /ar.gaz/ (box), is represented in the bottom-right corner. The words /si.ra/ and /max.∫ev/ (boat and computer, respectively) are unrelated distractor.

To specifically gauge the cognitive mechanisms involved in speech processing under adverse conditions, our laboratory adapted the *Visual World* eye-tracking paradigm [51] to include a concurrent task (increasing memory load) and noise (increasing distractions), creating the E-WINDMIL [44]. In E-WINDMIL, listeners hear Hebrew sentences such as “point at the box” while viewing a visual display on a computer screen that contains 4 objects. In this example (Figure 1), the display shows a picture of the named object heard by the participant, *box /ar.gaz/*, along with three other objects: a phonological competitor (eg, an onset competitor that shares the first syllable with the target, *rabbit /ar.nav/*) and 2 additional objects that are neither semantically nor phonologically related to the heard target object or its name. Participants are asked to touch the picture of the object as quickly and accurately as possible while their eye gaze is recorded. Rather than analyzing the slower overt touch response, only the eye gaze is taken into account in later analysis.

As real-life speech processing is often accompanied by other tasks, before the onset of the spoken instructions, participants are also asked to retain in memory either 1 or 4 digits (low or high memory load, respectively) for later recall. A discrimination score, which is the difference between the proportion of eye gaze fixations to the target (image representing the heard word) and the phonological competitor, was used to assess the 2 groups. The higher the difference, the more efficient the listener is in discriminating the spoken target from its signal-sharing competitor. Using the same eye-tracking paradigm, Nitsan et al [52,53] showed that listeners with larger WM capacity were able to identify the target word (and reject the signal-sharing competitor) earlier than a matched group with lower capacity. These findings suggest that improving one’s cognitive capacity might improve speech processing in adverse conditions.

### This Study

A total of 2 groups of older adults were tested *twice* on the E-WINDMIL speech processing task. One group received no cognitive training, whereas the other group followed the *Effectivate* SGCTA training protocol for 6 weeks. We aimed to test whether a short training period using *Effectivate* would engender a significant far-transfer change in speech processing ability. As the tested SGCTA does not involve any type of auditory training, improved performance on the E-WINDMIL speech processing task would provide strong support for the far-transfer effect and demonstrate the use of far-transfer measures in gauging training success for the validation of SGCTAs.

We hypothesized that if the tested SGCTA, *Effectivate*, improves generalized EFs, speech processing in adverse conditions, as measured by performance on the E-WINDMIL, would improve for the training group. Specifically, the difference in discrimination scores between the training group and control group would not be significant in the first test session, although an advantage for the training group would be found in the second test session (after training). This would suggest that training had a significant impact on real-time speech processing, above and beyond practice with the E-WINDMIL task.

## Methods

### Participants

A total of 54 older adults were recruited via phone calls from the Reichman University’s older adult research volunteer group and randomly assigned to either the cognitive training or control group. Although the groups cannot be said to reflect the diversity of the global older adult population, they are representative of the population residing in central Israel, where the study was conducted. Of these 54 individuals, 8 (15%) did not return for the second eye-tracking session, and 6 (11%) were excluded because of failure in eye movement recording or loss of eye-tracking signal. Recruitment was continuous for the duration of 6 months. Owing to the COVID-19 pandemic, participant recruitment and data collection were limited and terminated earlier than expected. The training group comprised 50% (20/40) of older adults (mean age 65.65, SD 4.8 years; 14/20, 70% were women). The control group comprised 50% (20/40) of older adults (mean age 69.05, SD 3.8 years; 13/20, 65% were women) from the study by Baharav et al [54]. All participants met the research inclusion criteria (refer to Textbox 1 for details). As shown in Table 1, the 2 groups had similar gender distribution (*P*=.74). Hearing acuity (pure tone average), years of education, and forward digit span scores did not differ significantly between the 2 groups (*P*=.51, *P*=.74, and *P*=.76, respectively). However, participants in the training group were slightly younger (*t*_38_=2.48; *P*=.02). All the participants provided written informed consent.

**Inclusion criteria for participant recruitment.**

**Language background**

High proficiency Hebrew speakers (no early bilinguals were included), assessed by a self-report and a score within the normal range in the Wechsler Adult Intelligence Scale–3 Hebrew vocabulary subtest

**Hearing**

Symmetrical air conduction hearing thresholds, expressed as pure tone averages of ≤25 dB hearing level in each ear (0.5 kHz, 1 kHz, and 2 kHz), and no reported history of auditory pathology

**Vision**

Normal or corrected to normal visual acuity and color vision, assessed by the Landolt C charts and the Ishihara charts

**Cognition: working memory**

Clinically normal scores for their age range on the Montreal Cognitive Assessment cognitive screening test and on the forward and backward digit span subtests (Hebrew version of Wechsler Adult Intelligence Scale–3 [46])

**Table 1:** Demographic characteristics (N=40).

Characteristics	Training group (n=20)	Control group (n=20)	Group comparison		
			*t* test^a^ (*df*)	Chi-square (*df*)	*P* value
Age (years), mean (SD)	65.65 (4.848)	69.041 (3.605)	2.478 (38)	N/A^b^	.02
Gender (women), n (%)	14 (70)	13 (65)	N/A	0.4 (1)	.74
Hearing (across 0.5 kHz, 1 kHz, and 2 kHz), mean (SD)	16.79 (4.939)	17.85 (4.913)	0.672 (37)	N/A	.51
Education (years), mean (SD)	16.42 (2.244)	16.18 (2.69)	0.339 (34)	N/A	.74
Digit span, mean (SD)	9.9 (1.714)	9.75 (1.333)	0.309 (38)	N/A	.76

^a^The *t* test was 2-tailed.

^b^N/A: not applicable.

### Ethics Approval

Ethics approval for this study was obtained from the Reichman University (Interdisciplinary Center Herzliya) institutional review board (P_1920119). This study was conducted in line with the CONSORT-EHEALTH (Consolidated Standards of Reporting Trials of Electronic and Mobile Health Applications and Online Telehealth) checklist (Multimedia Appendix 1).

### Stimuli

#### Auditory Stimuli

Auditory stimuli were taken from the study by Nitsan et al [52,53] and contained both the object names describing the visual stimuli and the sentence, “point at the ___ [target word]” in Hebrew using a plural non–gender-specific form. All object names were disyllabic. The average target word duration, including the Hebrew definition article *ha-* (the), was 1078 (SD 91) milliseconds. The root mean square intensity was equated across all recorded sentences. Files were mixed with a continuous speech spectrum noise at a fixed 0 dB signal-to-noise ratio based on values for the discrimination timeline in the study by Ben-David et al [55]. Stimuli were presented binaurally at 50 dB above the individual pure tone average via a MAICO MA-51 (MAICO) audiometer using TDH 39 supra-aural headphones (Telephonics).

#### Visual Display

In each trial, the participants were presented with a 3×3 grid with 4 images of objects positioned at the grid corners (Figure 1). The stimuli (images) were previously used in the studies by Hadar et al [56] and Nitsan et al [52,53] and were confirmed to be clearly identifiable and highly familiar. In all the trials, 1 of the 4 image names corresponded with the spoken target word. In critical trials, a second image name was a phonological competitor: sharing the initial syllable (onset overlap) or the final syllable (offset overlap) with the spoken target word. The remaining 2 objects presented on the screen were phonologically and semantically unrelated to both the target word and phonological competitor. In addition to critical trials, filler trials were used to diminish participant expectation of onset phonetic resemblance between the depicted object names. In filler trials, all 3 distractors were phonologically and semantically unrelated to the target word.

The original image database was divided into two to create 2 image sets, which were counterbalanced between participants for testing sessions 1 and 2. Within each testing session, objects were presented twice: once as a critical trial and once as a filler trial in which one of the 2 phonetically *unrelated* items was used as the target word. To prevent implicit spatial learning within a single testing session, object positions on the screen were rotated at each presentation.

### Procedure

#### Overview

The study comprised 2 experimental sessions, all conducted individually in a dedicated experimental laboratory complex at the Reichman University. In the first session, participants signed an informed consent form, and the inclusion criteria measures were collected. The E-WINDMIL paradigm was administered (as presented in the following section) to determine the participants’ baseline performance. To maintain experimenter blindness to the conditions, 2 different research assistants conducted the experiment. One research assistant conducted the E-WINDMIL and auditory testing, and the other research assistant assigned participants to each group and presented the participants in the training group with a web address providing access to the *Effectivate* SGCTA and instructed them to train at least three times a week for a duration of 5 weeks, after which they returned for the second experimental session. They were called once a week to verify *Effectivate* training. In the control group, participants were asked to maintain their daily routine and return within 2 to 4 weeks. In the second session, the same E-WINDMIL task was administered and the participants were debriefed. All participants were aware of the academic affiliation of the researchers. Participants in the experimental condition were not blinded to the name of the SGCTA company; however, the product was still in the beta stages and, as such, was not publicly available or marketed at the time.

#### E-WINDMIL Paradigm

The experiment was administered individually in a dedicated sound-attenuated booth (IAC Acoustics). Participants were seated 60 cm away from the computer screen, with their heads placed on the designated eye-tracker chin rest to minimize head movement. Each participant’s dominant eye was calibrated to ensure that their real-time eye gaze position was recorded throughout the course of the trial. A table-mounted SR Eyelink 1000 eye tracker (SR Research Ltd) in the *tower mount* configuration was used. Eye gaze position was recorded using the Eyelink software at a rate of 500 Hz.

Trials began with a visual cue of a black *play* triangle centered on the screen, immediately followed by the auditory presentation of either 1-digit preload (low-load condition) or 4-digit preload (high-load condition) through headphones. Participants were told to memorize these digits (in the order presented) for later recall. Subsequently, a 3×3 grid with the 4 images appeared (Figure 1). Participants were given 2 seconds to view the object positions, after which a fixation cross appeared in the center of the screen. Once the participants pressed the fixation cross to initiate the trial, the instruction sentence, “point at the ___ [target word],” was presented via the headphones. Selection of a named object was indicated by touching the object’s picture on the touch screen. Following the participant’s selection of a stimulus, a visual feedback signal appeared in the square of the selected image: red highlight for an incorrect answer or green highlight for a correct answer. Finally, the visual display was cleared, and a visual cue of a black circle appeared on the screen, signaling participants to recall aloud the digit preload from the beginning of the trial. Then, the experimenter coded the response (either correct or incorrect) in real time. Participants were instructed that the speed and accuracy of both the object selection and digit recall were equally important.

In a given testing session, participants completed 68 trials, split into 2 trial blocks for each digit preload condition (low load: 1 digit; high load: 4 digits). Each condition contained 34 trials, of which 2 (6%) were practice trials, and 32 (94%) were experimental trials. The 32 trials in each condition were split such that 16 (50%) were *filler* trials, indicating that the target object’s name did not share any phonology with the surrounding objects, and 16 (50%) were *critical* trials, indicating that the target object’s name shared phonology with a surrounding object name. 50% (8/16) were phonological onset competitors (eg, */ar.nav/-/ar.gaz/*), and 50% (8/16) were phonological offset competitors (eg, /xalon/-/balon/).

Although participants in the experimental group were aware of the intervention, the *Visual World* covert eye-tracking design was found to account for participants trying to outperform in an overt choice of the target (eg, with a button press). In other words, participants cannot control eye gaze fixations toward the alternatives versus fixations toward the target once saccades have been initiated. Indeed, in the visual world paradigm, eye movements were affected by implicit task goals and relatively immune to intentions and social desirability [57-59].

#### SGCTA Effectivate

**Figure 2:**
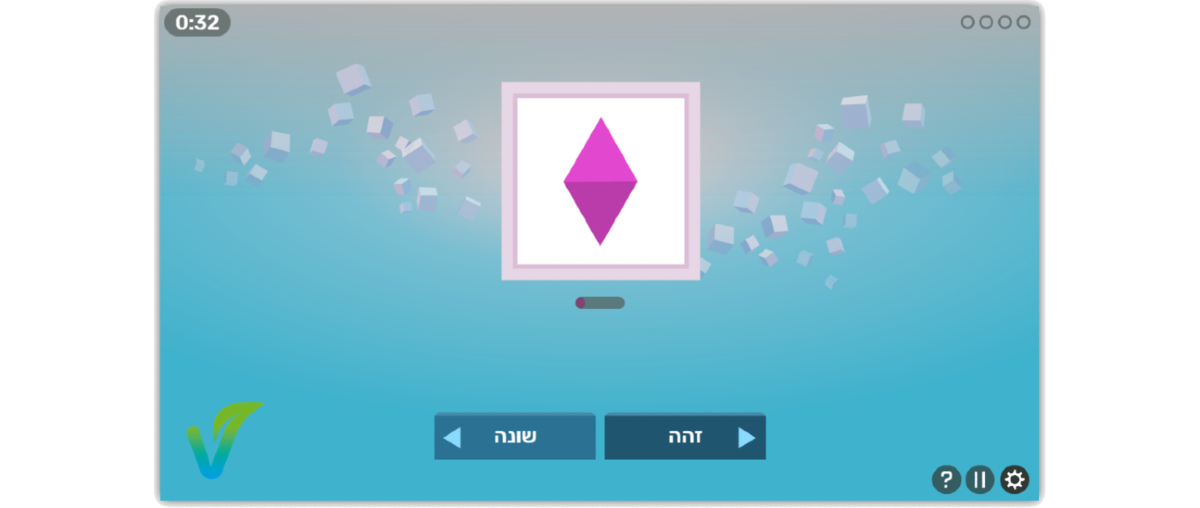
An example of a slide from the Effectivate serious game for cognitive training in aging—the exercise, The Last One Counts, is based on the ‘n-back’ task. In this exercise, the users were presented with a sequence of shapes and asked to decide whether each shape is identical to the one previously presented. Task difficulty changed gradually by updating different parameters, such as exposure latencies. In advanced levels, users were asked to decide whether the current shape is the same as, different from, or partially similar to the previously presented one. This additional level of complexity requires users to segregate the item’s different features (ie, color and shape) to selectively focus on some and inhibit others.

Following baseline testing, participants in the training group completed at-home web-based training, using a PC or tablet. A minimum of 15 training sessions were completed with approximately 8 minutes of active training per session (range 3-15 minutes). Each training session comprised 2 to 10 exercises, which were selected from a bank of 10 tasks. The difficulty level was individually adjusted for each participant and calibrated separately for each task using various measures (eg, exposure time, reaction time window, and number of objects). Each training task targeted at least one of the following cognitive functions: processing speed, WM, executive control, attentional control, sustained attention, spatial attention, binding, semantic memory, and training of several mnemonic methods. Figure 2 presents an example of such an exercise.

## Results

### Response Accuracy

Table 2 presents the accuracy percentage for each experimental condition—the percentage of trials in which participants both correctly selected the corresponding object on the visual display (indicating correct spoken word recognition) and correctly recalled the preload digits (indicating correct digit recall). A Mann-Whitney independent-sample nonparametric test confirmed that WM load, test session, and participant group did not have significant effects on accuracy, with *P*>.17 for all 4 tests.

**Table 2:** Mean percentage (and SDs) of trials in which the target word was correctly selected and digits were correctly recalled^a^.

Participant group and WM^b^ load			First session (%), mean (SD)		Second session (%), mean (SD)
**Training**					
	Low	99.4 (2.8)		98.1 (4.6)	
	High	91.2 (14.1)		88.1 (15.4)	
**Control**					
	Low	98.9 (3.7)		99.4 (4.6)	
	High	87.5 (13.4)		92.6 (10.7)	

^a^Low working memory and high working memory indicate the two preload conditions, 1 digit and 4 digits, respectively.

^b^WM: working memory.

### Eye Gaze Analysis

**Figure 3:**
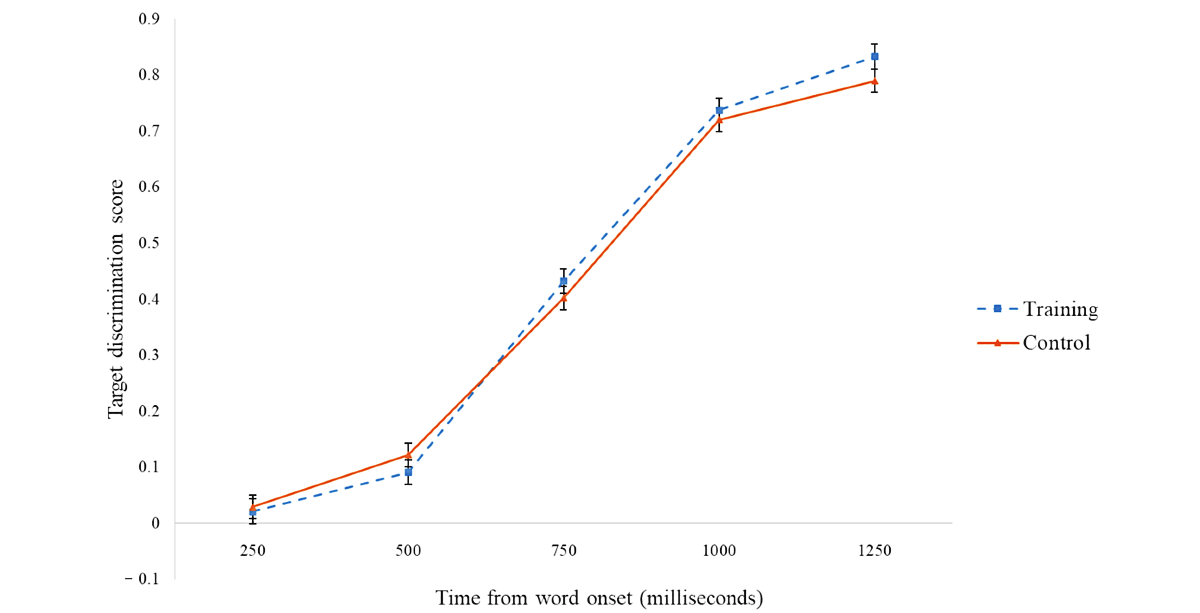
First test session. Mean target discrimination scores (with SE bars) for the training and control groups. Target discrimination scores are the proportion of fixations on the competitor subtracted from the proportion of fixations on the target within 250-millisecond time bins, starting from 250 milliseconds after the word onset to 1500 milliseconds.

**Figure 4:**
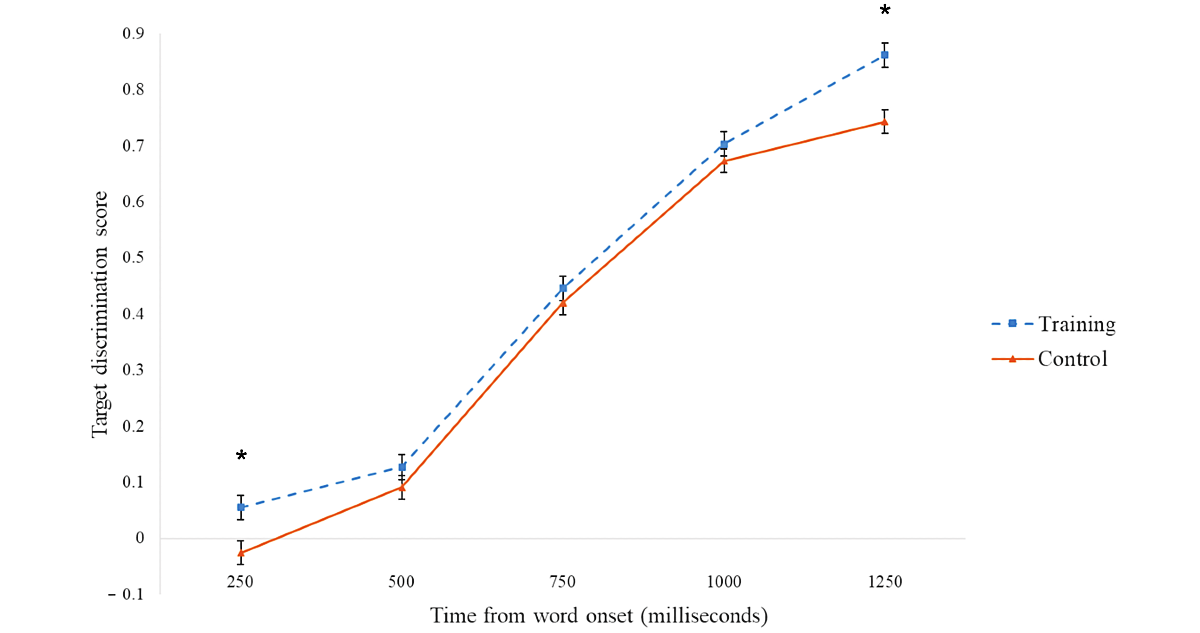
Second test session. Mean target discrimination scores (with SE bars) for the training and control groups. Target discrimination scores are the proportion of fixations on the competitor subtracted from the proportion of fixations on the target within 250-millisecond time bins, starting from 250 milliseconds after the word onset to 1500 milliseconds. *Significant effect.

We analyzed target discrimination scores (following the methodology of previous studies [60-63]) reflecting the listeners’ ability to discriminate the target word from its phonological competitor. The proportion of fixations on the competitor was subtracted from the proportion of fixations on the target within 250-millisecond time bins, starting from 250 milliseconds after the word onset to 1500 milliseconds. In this measure, the higher the value, the better listeners can discriminate the target from its competitor; values approaching 0 reflect an inability to discriminate between the target and competitor objects. Mixed-design repeated-measures ANOVAs were conducted for each 250-millisecond time bin, with three within-participants factors—WM load (high and low), test session (first and second), and condition (onset vs offset sound sharing)—and one between-participant factor—participant group (training and control). In each analysis, planned comparisons compared the effect of the participant group on discrimination scores in the first and second test sessions to verify whether differences between groups were related to the intervention (ie, significant effect only in the second session). Significant interactions of the test session with the participant group were noted in two of the five tested time bins: early processing 250 to 500 milliseconds and late processing 1250 to 1500 milliseconds, as discussed in the following section (Figures 3 and 4). The remaining three time bins (500-750 milliseconds, 750-1000 milliseconds, and 1000-1250 milliseconds) did not show any significant interaction; thus, they will not be discussed further.

### Early Processing: 250- to 500-Millisecond Time Bin

The interaction between the test session and the participant group was found to be approaching significance (*F*_1,38_=3.881; *P*=.06; *η*_p_^2^=0.093). Planned comparison indicated no significant difference between the 2 groups in the first session (*F*_1,38_=0.056; *P*=.81), whereas the second session produced higher discrimination scores in the training group (*F*_1,38_=7.371; *P*=.01; *η*_p_^2^=0.162). This suggests that improved performance can be related to the intervention itself. The effect of the participant group was marginally significant (*F*_1,38_=3.048; *P*=.07; *η*_p_^2^=0.085), with slightly higher discrimination scores in the training group (as in the previous analysis, this difference can be related to the second session) and no significant main effect for test session (*F*_1,38_=0.224; *P*=.64). No significant triple interactions were found for the participant group, test session, or any other tested variables (WM load or condition).

### Late Processing: 1250- to 1500-Millisecond Time Bin

A significant interaction between the test session and the participant group was found (*F*_1,38_=4.220; *P*<.05; *η*_p_^2^=0.100). Planned comparisons indicated that although the 2 groups did not significantly differ in the first session (*F*_1,38_=1.689; *P*=.20), the second session showed higher discrimination scores in the training group than in the control group (*F*_1,38_=9.003; *P*=.005; *η*_p_^2^=0.192), suggesting that improved performance could be related to the intervention itself. A significant main effect of the participant group was noted (*F*_1,38_=6.722; *P*=.01; *η*_p_^2^=0.150), with higher discrimination scores in the training group (emanating from higher scores in the second session) and no significant main effect for the test session (*F*_1,38_=0.108; *P*=.74). No significant triple interactions were found for the participant group, test session, or any other tested variables (WM load or condition).

In summary, in early and late processing (250-500 milliseconds and 1250-1500 milliseconds after word onset, respectively), performance did not differ between the 2 groups in the first test session. However, in the second test session after training with the *Effectivate* SGCTA, the training group surpassed the control group. These effects can be taken to suggest that the SGCTA training improved performance, over and above any effect of test-retest repetition.

## Discussion

### Principal Findings

In this exploratory study, we aimed to validate an eye-tracking paradigm, the E-WINDMIL, which tests real-time speech processing in adverse conditions as a gauge for the far-transfer efficacy of SGCTAs. Specifically, we tested whether training EFs in the visual modality with the SGCTA *Effectivate* generalizes to improved speech processing in adverse listening conditions (auditory modality) for older adults.

The training group, with 50% (20/40) of the older adults, was tested before and after 6 weeks of training on the SGCTA *Effectivate*. The control group, with another 50% (20/40) of the older adults, did not undergo any specific cognitive training. Before training, no significant differences in E-WINDMIL performance were noted between the control group and the training group. However, after training with *Effectivate,* the training group outperformed the control group in early word processing (indicated by eye movements, 250-500 milliseconds after word onset) and late word processing (1250-1500 milliseconds after word onset). The early processing advantage may suggest improved stream segregation between the spoken target word and noise [52,53], when WM was otherwise occupied. The late processing advantage alludes to improved decision-making processes (ie, using accumulated evidence) once the word had been completely heard [55]. Our results provide early support for the efficacy of the E-WINDMIL speech processing paradigm as a far-transfer measure of cognitive training with the SGCTA *Effectivate*. This is of special interest as the tested SGCTA did not train any auditory task or spoken language processing.

### Speech Processing as a Far-Transfer Gauge for Cognitive Training

Challenges in determining the effectiveness of any cognitive intervention stem from the ongoing debate: Do we use near-transfer or far-transfer metrics? [14] In other words, is it sufficient to indicate improved performance on the trained task or should research indicate improved performance on a *daily* task, far from training, to suggest the *generalizability* of training? This exploratory study demonstrates the efficacy of using a far-transfer measure that involves speech processing in adverse conditions to discern the impact EF training has on daily life activities.

Speech processing in adverse conditions presents an excellent gauge of the generalizability of cognitive training. As speech processing is resource demanding, the fewer resources listeners have, the more they will be affected by adverse conditions such as background noise. Speech processing involves holding ongoing speech strings in memory and integrating words and phrases to create coherent meaning; thus, it is considered to be dependent on WM and other attentional resources [52,56]. The results of this study suggest the prowess of training to create a generalized cognitive effect, as a few weeks of training on the *Effectivate* SGCTA was sufficient to improve speech processing in adverse conditions (above and beyond test-retest learning effects).

This improvement can be interpreted in light of the crucial role of EFs, especially WM, in speech processing in adverse conditions. According to the Ease of Language Understanding model [44], explicit WM resources are drawn from a central pool to compensate for the loss of automatic matching between the input and lexical representations when the sound input is degraded by adverse listening conditions. Other studies have demonstrated a direct link between WM capacity and the ability to inhibit irrelevant information. This ability is necessary to separate the speech signal from background noise and reject competing words in the mental lexicon. Thus, our results suggest that training EFs using SGCTAs might have a generalized effect on real-life daily tasks. Returning to our example in the introduction, with improved WM capacity, the older adult will be better able to understand his grandson saying, “Grandpa, have you seen the DOLL?” rather than *DOG* (sound-sharing alternative) while driving a car (WM load) with the radio playing (adverse listening conditions) in the following ways:

1. Improve speech segregation—separating the spoken message from the background task-irrelevant noise (eg, radio and engine noise)
2. Effectively inhibit the activation of competing similar-sounding words in the mental lexicon (eg, *DOG*)
3. Allocate enough resources to use context and information in long and short memory from a cognitive resource pool, which is already depleted by the concurrent task of driving

Given the pivotal role of speech processing in successful aging [64], this change may have a lasting positive impact on the quality of life in older age.

### E-WINDMIL as a Far-Transfer Gauge for Cognitive Training

The advantage of using the adapted visual world paradigm E-WINDMIL lies in its increased ecological validity—measuring a daily task (speech processing) that is important to the perseverance of well-being and performance in older age [17-19]. Eye tracking used by E-WINDMIL is better suited to test older adults’ speech processing than more traditional speech tests involving overt responses, such as verbal or keypress response. It is not influenced by an age-related slowing of motor speed, which often affects non–real-time speech tests [65]. Unlike many other speech processing tasks that assess the processing of a single word in *ideal listening* conditions, the E-WINDMIL asks listeners to retain digits for later recall (a task designed to weigh on WM resources) while presenting speech in noise. In this way, the E-WINDMIL paradigm acknowledges that speech in real-life scenarios is often experienced along with noise while the listener is engaged in other cognitively demanding tasks (eg, following the context of the sentence as it unfolds and driving). Moreover, eye tracking has been shown to be a sensitive measure for speech processing in various studies, suggesting that speech processing is costly in terms of WM processing and perhaps even mediated by it [29,34,56,66,67].

### Training-Related Advantage in the 1250- to 1500-Millisecond Time Bin

Previous adaptations of E-WINDMIL found eye tracking to be very sensitive to differences in cognitive reserve. Hadar et al [56] found that minimizing available cognitive resources can slow down processing in this task. Nitsan et al [52,53] found that individuals with higher cognitive reserve outperform individuals with lower reserve while using E-WINDMIL. This advantage, attributed by the authors to the use of cognitive resources for speech processing, was indicated in the later time bins—similar to the current findings. Other studies also found that differences in cognition were indicated in later word processing with older listeners in particular [61,68]. A recent study by Harel-Arbeli et al [46] attributed the advantages seen in later time bins to decision-making processes. In their study, using a similar eye-tracking paradigm, the spoken target word was preceded by a spoken predictive context presented in a quiet environment. An advantage of young adults over older adults, based on the age-related difference in cognitive resources, was present mainly in the late time bin when the full word had been spoken. Taken together, it appears that improved processing in the late time bin may reflect improved cognitive resources (eg, WM and inhibition).

### Training-Related Advantage in the 250- to 500-Millisecond Time Bin

The current data also indicated training-related advantages in processing during early time bins, when only the first phoneme of the word is being processed. This suggests that cognitive training improved target word stream formation and auditory stream segregation between the target word and noise [55,69]. Indeed, this early process of stream segregation has been linked not only to sensory processes but also to the deployment of cognitive resources. Cognition is necessary for the inhibition of the noise stream and selective focus on the target word stream, leading to stream segregation [70,71]. Stream segregation is essential for speech processing and represents one of the major hurdles for older adults in social interactions [72]. Indeed, age-related auditory sensory degradation can specifically impair processes related to stream segregation in aging [73]. This early time bin training advantage may also be related to the early benefits noted in the literature as a result of removing background noise [60] and increasing the lexical frequency of the spoken word [74] using similar eye-tracking paradigms. In summary, the performance advantage in the early time bin associated with SGCTA training may reflect an increase in cognitive reserve.

### Caveats and Future Studies

This study should be taken as a first step in supporting the effectiveness of the tested SGCTA, and it does not serve as a recommendation or suggestion to use SGCTAs in general or specifically the *Effectivate* SGCTA. This study was ongoing at the beginning of the COVID-19 pandemic and was halted because of national quarantine. Therefore, we were unable to amass a larger group of participants. Moreover, we were unable to recruit an active control group to undergo an alternative form of cognitive training. Future studies should attempt to replicate the results with an active control to ensure that the observed effects were not related to possible social desirability or lack of participant blinding but to the specific cognitive training, *Effectivate*. However, we note that the experimenters administering the study were blinded to the condition, and the experimental tool was relatively immune to social desirability. Such replications should also more carefully match participants across all groups. Indeed, on average, participants in the control group were older by a few years than those in the training group. We also note that participants in this study did not form a representative sample of the older adult population, specifically given the cognitive and linguistic inclusion criteria. Although future studies should aim to include more diverse samples, these criteria are common in research with this population [75-77].

We demonstrated that the *Effectivate* SGCTA is sufficiently powerful to induce changes, even in cognitively healthy older adults, and that the E-WINDMIL test is sufficiently sensitive to detect such changes. Our preliminary results are the first step, suggesting the ability of the SGCTA *Effectivate* to engender far cognitive transfer. Future studies should also try to relate our results to other more traditional cognitive measures and questionnaires tapping users’ subjective evaluation of their quality of life.

### Summary and Implications

This exploratory study presents an early foray into the potential of speech processing in adverse conditions as a far-transfer gauge of SGCTAs. This is in line with previous studies that used gamification in cognitive decline research [78-81]. Results present a preliminary indicator of the SGCTA *Effectivate’s* potential to engender such far transfer from visual cognitive training to auditory speech processing after only a few weeks of training. Following training, older adults were better able to differentiate between the spoken target word and its sound-sharing competitor under adverse conditions (noise and digit memorization). We suggest that this change in performance represents a real-world improvement in a daily task that is directly related to successful aging. Thus, it shows the potential of the training to have a significant impact on the user’s daily life. We advocate that cognitive training should showcase evidence-based improvement in daily far-transfer tasks that can change the user’s quality of life, as opposed to merely showing changes in traditional pen-and-paper cognitive measures. As serious games are a means of improving performance in other tasks, games developed to the highest standards should seek out far-transfer validation methods. We hope that the increased demand for far-transfer metrics will bolster research efforts within the academic community to develop new far-transfer gauges of cognitive ability and call on serious game developers to adopt far-transfer metrics, such as E-WINDMIL, into their gauges for validity and success.

This study investigated aging through the lens of speech processing, a novel vantage point, which can illuminate interconnected attentional mechanisms known to be affected by aging. Most importantly, speech processing is an essential daily task performed across social interactions, leisure, and employment [72]. Impaired speech processing may have severe implications for older adults across all aspects of life. Therefore, we encourage adding tests of speech processing, especially in adverse conditions, to the arsenal of tools used to test the efficacy of EF training in aging. Furthermore, we suggest paying attention to speech processing in aging when considering accessibility and inclusion in serious game design.

In addition to being a novel and important test metric for aging, real-time speech processing metrics may also prove beneficial to testing other populations such as children with the neurodevelopmental disorder, attention-deficit/hyperactivity disorder (ADHD). As the most prevalent neurodevelopmental disorder in children, ADHD is associated with lifelong impairment, with symptoms reflecting a deficit in EFs such as inhibitory control, attentional regulation, and WM [82,83]. Given ADHD’s high prevalence and detrimental effect on the quality of life and well-being, many serious games are being developed to train EFs in ADHD. As is the case with SGCTAs, there is much debate in the literature regarding their efficacy [84]. Expanding on our findings, we suggest further exploration using E-WINDMIL to test the far-transfer efficacy of serious computerized games designed for children and adults with ADHD along with other promising populations that could benefit. We hope that the creation and use of universally accepted far-transfer metrics will determine gold standard serious games that will help us prolong cognitive functions and improve well-being with age and throughout life.

## Appendix

Multimedia Appendix 1 CONSORT-eHEALTH checklist (V 1.6.1).

## Abbreviations

ADHD: attention-deficit/hyperactivity disorder
CONSORT-EHEALTH: Consolidated Standards of Reporting Trials of Electronic and Mobile Health Applications and Online Telehealth
E-WINDMIL: Eye tracking of Word Identification in Noise Under Memory Increased Load
EF: executive function
SGCTA: serious game for cognitive training in aging
WM: working memory
